# Diagnostic Value of MRI of the Sacroiliac Joints in Juvenile Spondyloarthritis

**DOI:** 10.5334/jbr-btr.1198

**Published:** 2016-11-19

**Authors:** Nele Herregods, Joke Dehoorne, Jacob Jaremko, Rik Joos, Xenofon Baraliakos, Koenraad Verstraete, Lennart Jans

**Affiliations:** 1 Ghent University Hospital, BE; 2 University of Alberta Hospital, CA; 3 Ruhr-University Bochum, DE

**Keywords:** Diagnostic value, Juvenile spondyloarthritis, Magnetic resonance imaging, Sacroiliac joints, Sacroiliitis

## Abstract

Early diagnosis of spondyloarthritis (SpA) is becoming more important as new medical treatment options have become available to treat inflammation and delay progression of the disease. Increasingly, magnetic resonance imaging (MRI) of the sacroiliac joints is obtained for early detection of inflammatory changes, as it shows active inflammatory and structural lesions of sacroiliitis long before radiographic changes become evident. MRI of the sacroiliac joints in children is a useful tool for suspected juvenile spondyloarthritis (JSpA), even though it is not yet included in the current pediatric classification systems.

Recognizing MRI features of pediatric sacroiliitis is a challenge. As most radiologists are not familiar with the normal MRI appearance of the pediatric sacroiliac joint, clear definitions are mandatory. Actually, the adult Assessment of Spondyloarthritis International Society (ASAS) definition for sacroiliitis needs some adaptations for children. A proposal for a possible pediatric-specific definition for active sacroiliitis on MRI is presented in this review. Furthermore, MRI without contrast administration is sufficient to identify bone marrow edema (BME), capsulitis, and retroarticular enthesitis as features of active sacroiliitis in JSpA. In selected cases, when high short tau inversion recovery (STIR) signal in the joint is the only finding, gadolinium-enhanced images may help to confirm the presence of synovitis.

Lastly, we found a high correlation between pelvic enthesitis and sacroiliitis on MRI of the sacroiliac joints in children. As pelvic enthesitis indicates active inflammation, it may play a role in assessment of the inflammatory status. Therefore, it should be carefully sought and noted when examining MRI of the sacroiliac joints in children.

## Introduction

The terms *spondylarthropathy, spondyloarthritis*, and *spondyloarthritides* (SpA) are used to refer to a group of related seronegative (i.e., rheumatoid-factor negative) inflammatory diseases characterized by enthesitis and arthritis [1,2,3]. Entheses are sites where tendons, ligaments, capsules, or fascia are attached to bone, providing a mechanism for reducing stress at the bony interface [4,5,6]. There is a strong association between SpA and the human leukocyte antigen (HLA) B27 [1]. The term *juvenile spondyloarthritis* (JSpA) refers to spondyloarthritis that starts during childhood (before age 16). Juvenile spondyloarthropathy (JSpA) represents an important subgroup of chronic arthritis in children that needs to be recognized and appropriately managed [1,2]. Although JSpA presents with peripheral arthritis and enthesitis in most cases, axial involvement may be more common than previously thought [7]. Axial inflammatory lesions have a tendency to ossify, resulting in ankylosis and contributing to a reduced quality of life and substantial disability [2]. Identification of those children is critical, as early screening and treatment may significantly impact the disease’s course and delay progression of the disease [7,8,9,10,11].

Historically, sacroiliac joints have been imaged by radiography; however, radiographic evidence of SpA is delayed and may under-represent active disease [8]. Radiographs can only demonstrate structural changes such as sclerosis, erosions, and ankylosis and cannot detect early active inflammation [12]. Increasingly, magnetic resonance imaging (MRI) of the sacroiliac (SI) joints is obtained for early detection of inflammatory changes [13], as active inflammatory and structural lesions of sacroiliitis can be seen long before radiographic changes become evident [13,14].

In adults, the presence of active sacroiliitis on MRI is a key criterion in the Assessment of Spondyloarthritis International Society (ASAS) classification [15,16,17]. In the adult ASAS criteria, there is a clear definition of a positive MRI for sacroiliitis [15]. At the moment, no such definition of a positive MRI for sacroiliitis exists in children with JSpA.

Understanding the MRI findings in pediatric sacroiliac joints is challenging, and given the potential lifelong morbidity of a missed diagnosis, building up experience in this specific topic is important. Furthermore, there is no clear definition of a “positive” MRI of the sacroiliac joint in JSpA. As most radiologists are not familiar with the normal MRI appearance of the pediatric sacroiliac joint, good definitions are mandatory to make a correct diagnosis of sacroiliitis in children.

Recently, some studies emphasized the usefulness of MRI in juvenile arthritides [13,14,18,19,20]; however, in children, the diagnostic value of MRI assessment of sacroiliitis has yet to be determined. The aim of our studies [21,22,23] was to provide more insight in the diagnostic value of MRI of the sacroiliac joints in children with or suspected for JSpA.

## Anatomy of the Sacroiliac Joint

**Figure 1:**
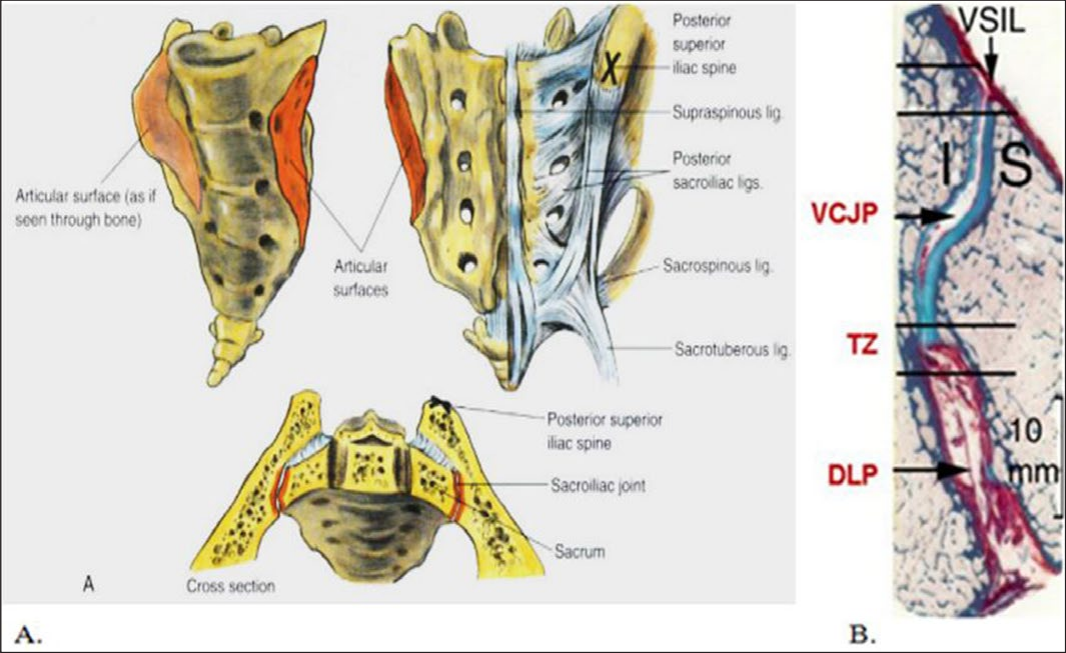
**a.** The anatomy of the sacroiliac joint, with the synovial part (red) and the ligamentous part (blue). (Brown atlas of regional anesthesia: /das/book/0/view/1353/I4-u1.0-B1-4160-2239-2..50044-8--f3.fig/top) **b.** Oblique transaxial histological section obtained through the middle third of the SIJ, with the iliac bone and cartilage (I) to the left and the sacral bone and cartilage (S) to the right side. The ventral cartilaginous (VCJP) and dorsal ligamentous portion (DLP) of the joint is separated by the dorsal transition zone (TZ) containing the dorsal 2 mm of the cartilaginous joint facets and the ventral 2 mm of the ligamentous joint space. The ventral sacroiliac ligament (VSIL) is also shown. A marker of 10 mm shows the true size of the section [22].

The sacroiliac joint is the joint in the bony pelvis between the sacrum and the ilium of the pelvis. The SI joint is a complex joint with two parts: the synovial part and the ligamentous part. The synovial part is located in the anterior and lower third of the joint. It is a synovial joint with hyaline cartilage on the joint surfaces surrounded with synovium. The ligamentous part is located in the dorsal and upper two-thirds of the joint, where the sacrum and the ilium are connected with restraining ligaments (anterior and posterior sacroiliac, interosseous, sacrotuberous, and sacrospinous ligaments) (Figure 1) [24]. In children, due to the natural growth process, the appearance of the sacroiliac joint is even more complex. There are significant age- and sex-related differences, as progressive ossification of the segmental and lateral apophyses of the sacral wings occurs [20]. This ossification process continues into late adolescence and is completed significantly earlier in girls than in boys [20].

## MRI Scan Protocol

**Figure 2:**
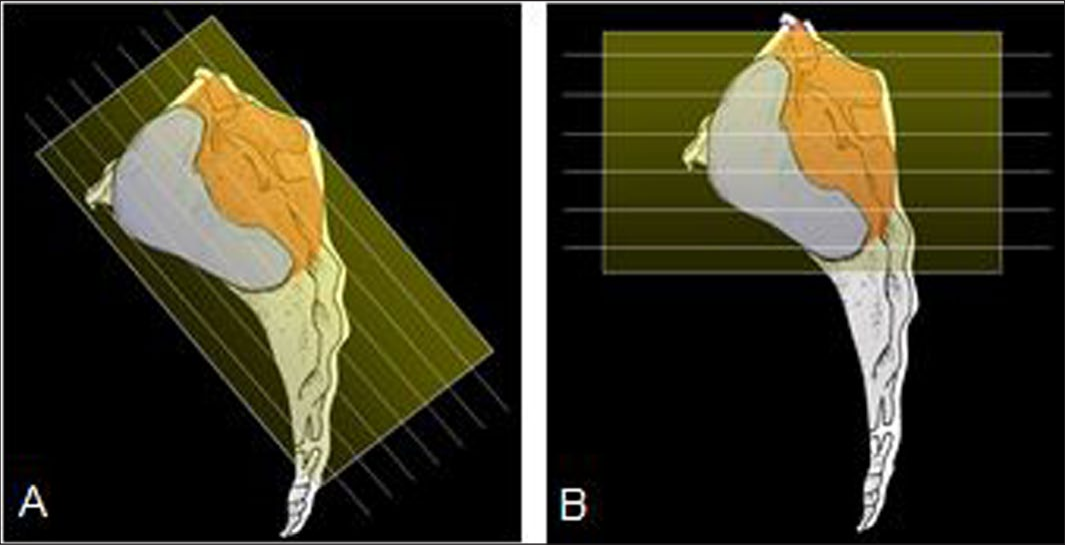
**a.** Paracoronal slice direction along the long axis of the sacrum, perpendicular to the second sacral (S2) vertebral body; **b.** Axial slice direction.

For the scan protocol of the sacroiliac joint in our studies [21,22,23], we used the classical/typical sequences obtained in adults (paracoronal T1-weighted and short tau inversion recovery (STIR) images and axial STIR images), complemented by contrast-enhanced (CE) fat-saturated axial and paracoronal T1-weighted sequences after intravenous administration of gadolinium (Gd) contrast. Paracoronal is defined as slice direction along the long axis of the sacrum, perpendicular to the second sacral (S2) vertebral body (Figure 2A). Obtaining axial images (Figure 2B) is important to get the whole picture. Next to the sacroiliac joint, the pelvic entheses and the hip joints can also be seen on these axial images. Hip joints are frequently involved in juvenile SpA, as JSpA in most cases presents with arthritis/enthesitis of the lower extremities [1,2,3]. Pelvic entheses can be seen as well and are important too, as enthesitis is another primary feature of JSpA, and the number of active entheses can predict sacroiliitis at follow-up [19].

**Figure 3:**
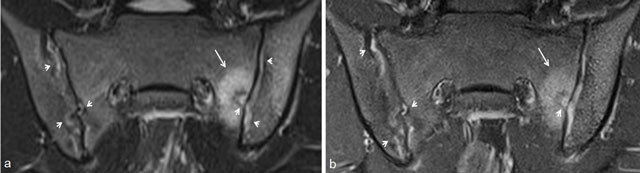
Active and structural sacroiliitis in a 17-year-old girl with JSpA: no added value of Gd. **a.** Semicoronal STIR image shows high signal in the joint space at both sides and in the erosions (small arrows) and BME on the left sacral side (large arrow). **b.** Contrast-enhanced fat-saturated T1-weighted image shows synovial enhancement in both SI joints in the erosions (small arrows) and enhancing osteitis on the left (large arrow).

**Figure 4:**
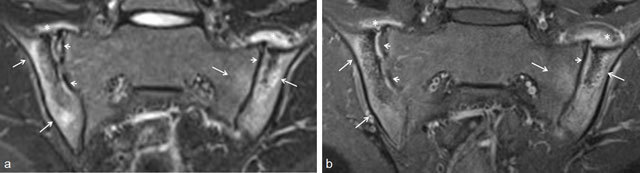
Active and structural sacroiliitis in a 16-year-old girl with JSpA: no added value of Gd. **a.** Semicoronal STIR image shows high signal in the joint space of both SI joints (small arrows) and bilateral BME (large arrows). Note also the bilateral extensive capsulitis (asterisk). **b.** Corresponding contrast-enhanced fat-saturated T1-weighted image shows synovial enhancement in both SI joints (short arrows), enhancing osteitis (arrows), and enhancing capsulitis on both sides (asterisk).

**Figure 5:**
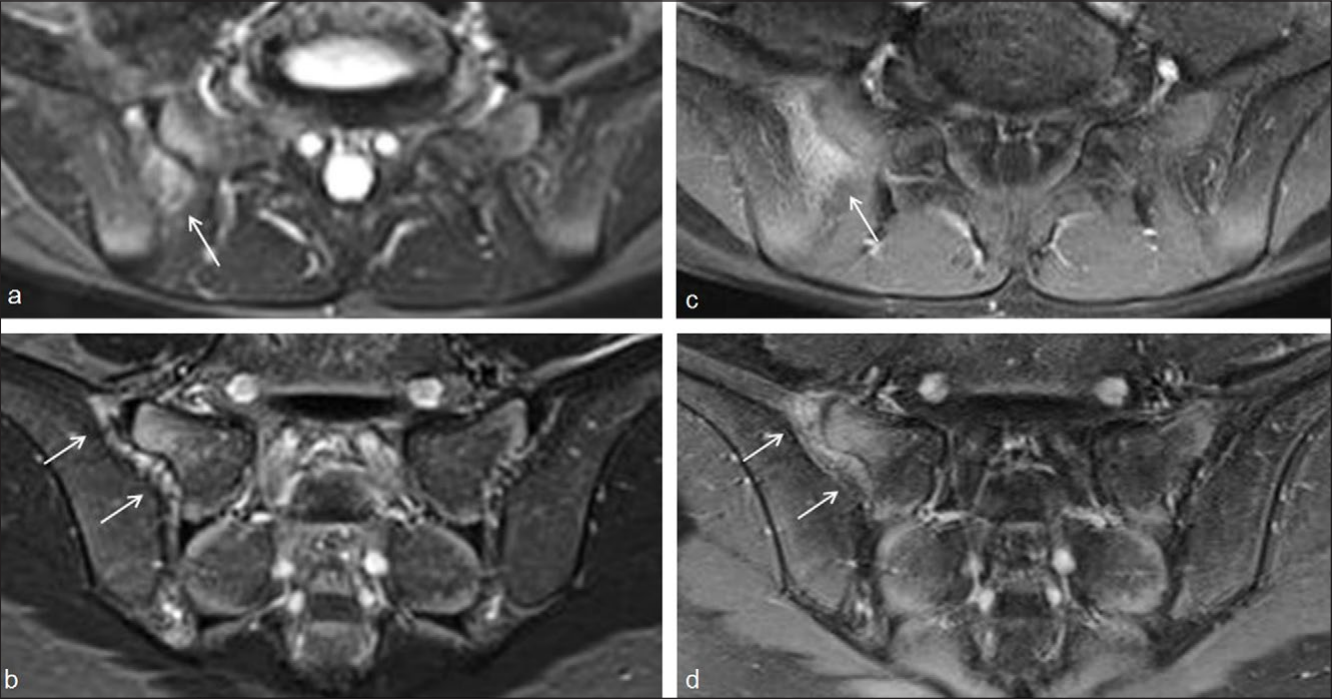
Active sacroiliitis with retroarticular enthesitis and capsulitis in a 14-year-old boy with JSpA: no added value of Gd. Axial and semicoronal STIR (**a–b**) and contrast-enhanced fat-saturated T1-weighted (**c–d**) images demonstrate enthesitis of the retroarticular interosseous ligaments and capsulitis on the right side (arrows).

**Figure 6:**
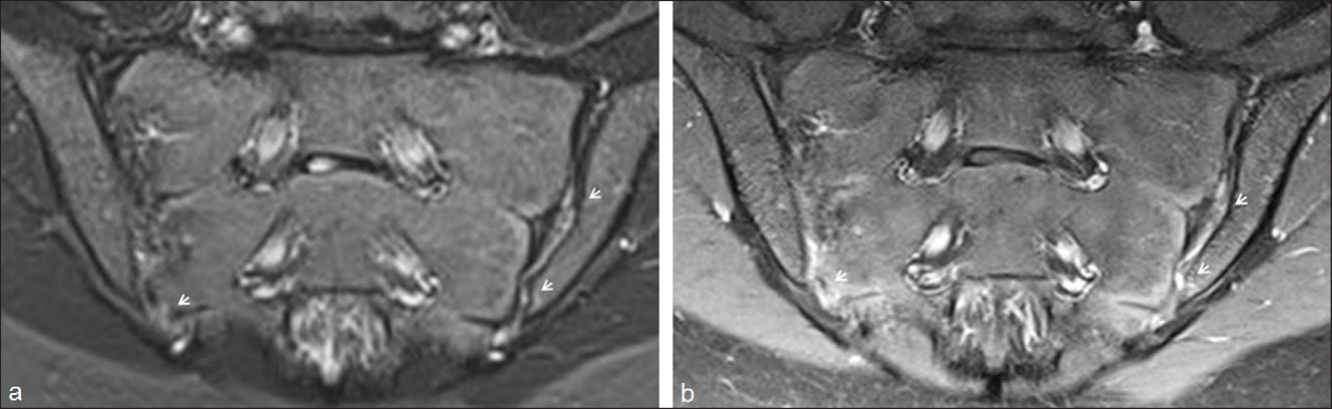
Synovitis representing active sacroiliitis in a 14-year-old girl with JSpA. **a.** Semicoronal STIR image shows high signal in the joint space of the left SI joint, discrete also in the caudal part of the right SI joint, with **b.** synovial enhancement representing synovitis in both SI joints on the contrast-enhanced fat-saturated T1-weighted image. No BME is seen.

In adults, it has been shown that contrast-enhanced (CE) sequences have no added value to diagnose active sacroiliitis [25,26,27,28]. But in children, this is not clear, so we included these CE-sequences in our scan protocol [21,22,23]. We showed that MRI without contrast (gadolinium (Gd)) administration is sufficient to identify bone marrow edema (BME), capsulitis, and retroarticular enthesitis as features of active sacroiliitis (Figures 3, 4, 5) [22]. CE-sequences are also mandatory to diagnose synovitis. When high signal in the joint space is the only MR finding, contrast-enhanced sequences are necessary to differentiate between fluid in the joint and synovitis, as both have high signal on T2/STIR images and contrast enhancement is only seen in synovitis (Figure 6) [15]. In our studies, we showed that synovitis in children is frequently present and—in contrast to adults—can be seen without accompanied bone marrow edema (BME) [21,22], confirming the findings of Lin et al. [18].

**Figure 7:**
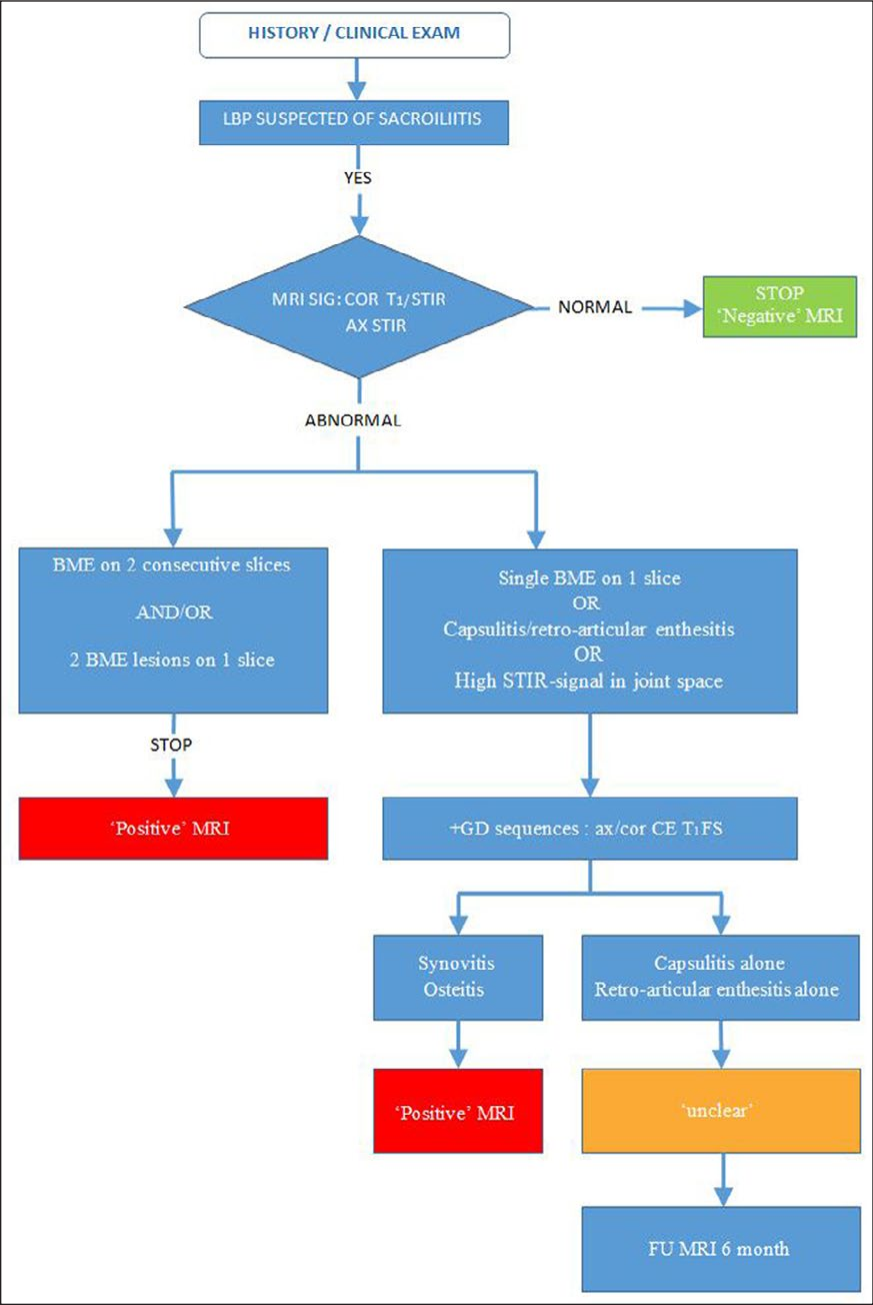
Flow chart for daily clinical practice for assessing sacroiliac joints on MRI in children (LBP = low back pain; MRI = magnetic resonance imaging; COR = coronal; STIR = short tau inversion recovery; AX = axial; BME = bone marrow edema; GD = gadolinium; CE= contrast-enhanced; FS = fat-saturated; FU = follow-up).

In children, we do not yet know whether fluid in the joint space represents a normal finding. Thus, in cases where synovitis is the only MR feature of active sacroiliitis, the diagnosis could be missed if no CE-sequences were included. In daily clinical practice, however, we would suggest including this CE-sequences, except for cases with clear presence of subchondral or periarticular BME on two consecutive slices or more than one lesion on a single slice. As it is not clear at the moment whether a BME lesion on a single slice, capsulitis or retroarticular enthesitis alone are sufficient to diagnose active sacroiliitis on MRI, we suggest to include CE-sequences in those cases to confirm osteitis or to diagnose concomitant synovitis. CE-sequences can make it easier to make a correct diagnosis with confidence, especially for radiologists who have less experience with reading MRI of the pediatric sacroiliac joints. A flowchart for daily clinical practice is presented for assessing sacroiliac joints on MRI in children (Figure 7).

## Image Review

Unlike adults, MRI assessment of sacroiliitis in children is not well studied. Our research demonstrates that MRI of the sacroiliac joints in children is a useful tool and, in our opinion, should be applied in clinical practice in all children suspected for juvenile spondyloarthritis (JSpA), even though it is not included in the current pediatric classification systems [21]. It can help in cases of a doubtful diagnosis and can identify patients with axial involvement, which has major implications for treatment decisions.

**Figure 8:**
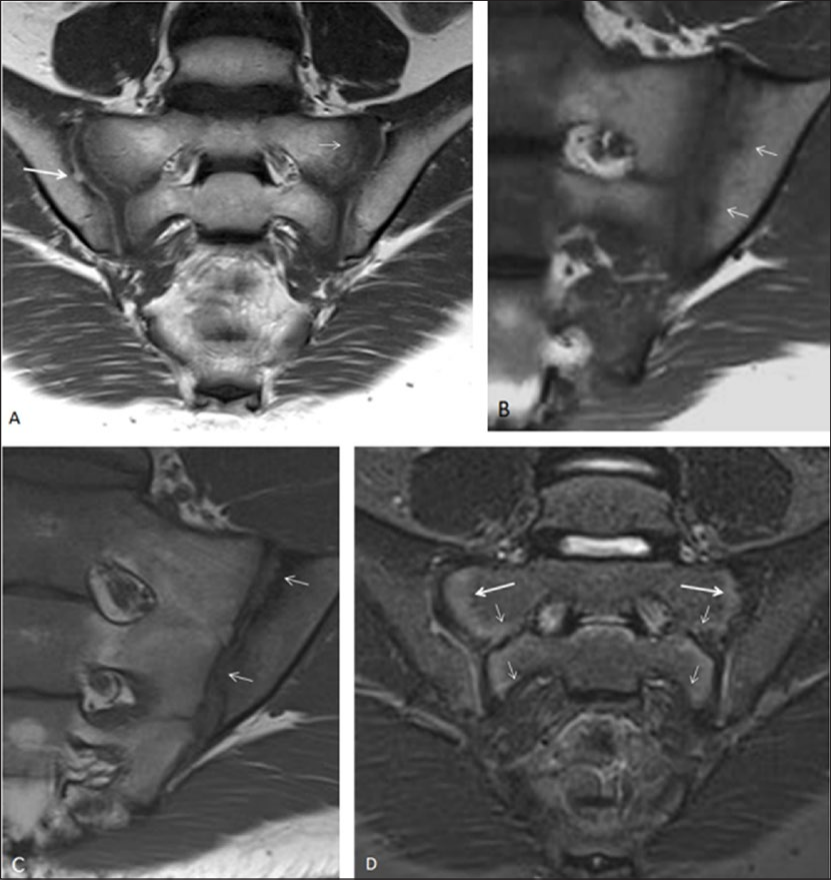
**a.** Paracoronal T1-weighted image of the sacroiliac joint, illustrating the ongoing ossification process, with open segmental and lateral apophyses of the sacral wing, resulting in a difficult delineation of the joint space due to the ongoing ossification process (small arrow). It is also very difficult to determine whether the nodular lesion in the right sacroiliac joint is an erosion (large arrow). **b–c.** Paracoronal T1-weighted images again illustrating the irregular outline of the developing sacroiliac joint, making assessment of erosions very hard. **d.** Paracoronal STIR image of the sacroiliac joint, illustrating the difficult assessment of bone marrow edema due to the high STIR signal of non-ossified cartilage in segmental (small arrows) and lateral (large arrows) apophyses.

MRI of the pediatric sacroiliac joint is challenging. Children are not small adults, and imaging features are definitely different in children compared to adults [21,29]. We must take into account the natural growth process of children with progressive ossification, which complicates the MRI assessment of the sacroiliac joint in children. Especially in smaller children, the segmental and lateral apophyses of the sacral wings are not yet ossified (Figure 8a), and the width of the joint space can vary depending on the age [20]. There is also a significant gender difference that makes interpreting these studies even more difficult [20]. As a result of this ossification process, the assessment of bone marrow edema and the delineation of the joint space can be notoriously difficult in children (Figure 8b–d). Non-ossified cartilage has a high signal on STIR [20], making it difficult to assess the presence of BME. We also noticed that the delineation of the joint can be very irregular and blurred.

In children, erosions can be better delineated on T2-weighted images, whereas in adults classically T1-weighted images are used for determining the presence of erosions. Bollow et al. also concluded that joint contours are more sharply delineated on T2-weighted images, irrespective of the children’s age and sex [18]. The assessment of erosions in children can therefore be difficult. In our research, we experienced that reading MRI of the sacroiliac joints in children has a steep learning curve. Next to the challenge of the complex, changing anatomy, there is also the relative lack of referrals for MRI of the sacroiliac joints in children, even in a tertiary care center.

## Features of Sacroiliitis

There are multiple features of active inflammation and structural damage (Table 1) visible on MRI of the sacroiliac joints that can provide a specific diagnosis of JSpA when present in children with suspected sacroiliitis [21].

**Table 1:** Typical Features of Sacroiliitis That Can Be Seen on MRI.

Typical MRI Features of Sacroiliitis*
Active inflammatory lesions – Bone marrow edema (BME) – Synovitis – Capsulitis – Retroarticular enthesitis
Structural/chronic lesions – Erosions – Sclerosis – Fat deposition – Ankylosis

* Adapted from Sieper J, Rudwaleit M, Baraliakos X, et al., 2009 [15].

**Figure 9:**
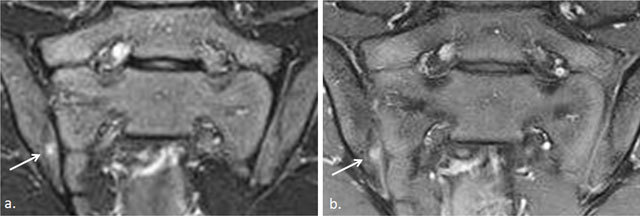
Active sacroiliitis in a 14-year-old boy with JSpA. **a.** Semicoronal STIR image shows a focal spot of BME at the iliac side of the right sacroiliac joint (arrow). **b.** Semicoronal contrast-enhanced fat-saturated T1-weighted image shows corresponding enhancement of this spot (arrow).

We found the MRI features of sacroiliitis in children to be slightly different compared to adults [21,29]. The most important features of active sacroiliitis in children are BME, synovitis, and capsulitis. In our experience, sacroiliitis in children often seems to be less extensive, with BME less pronounced. In some cases, only small, single-slice BME lesions are found (Figure 9), which may be important too and should be carefully sought-after and noted by radiologists. In some cases, capsulitis or synovitis can be seen with little or no surrounding edema; therefore, it is important to notice these features of active sacroiliitis, even when no BME is present [21].

**Figure 10:**
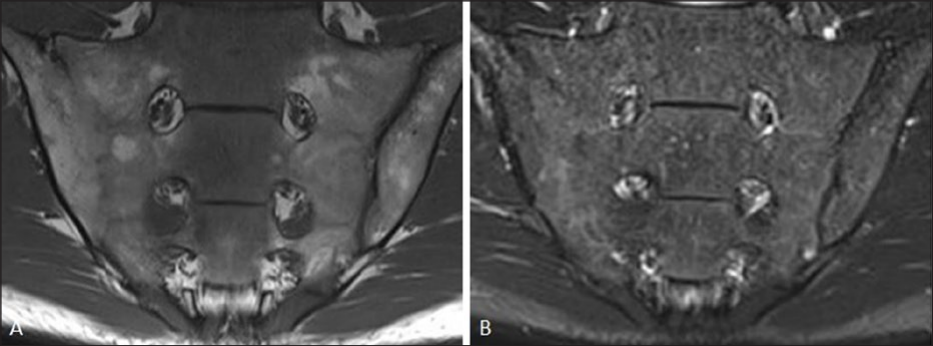
Ankylosis of the SI joint in an 18-year-old boy with JSpA. Semicoronal T1-weighted (**a**) and STIR (**b**) images show ankylosis of the right SI joint and narrowing of the left SI joint. No active lesions were seen.

**Figure 11:**
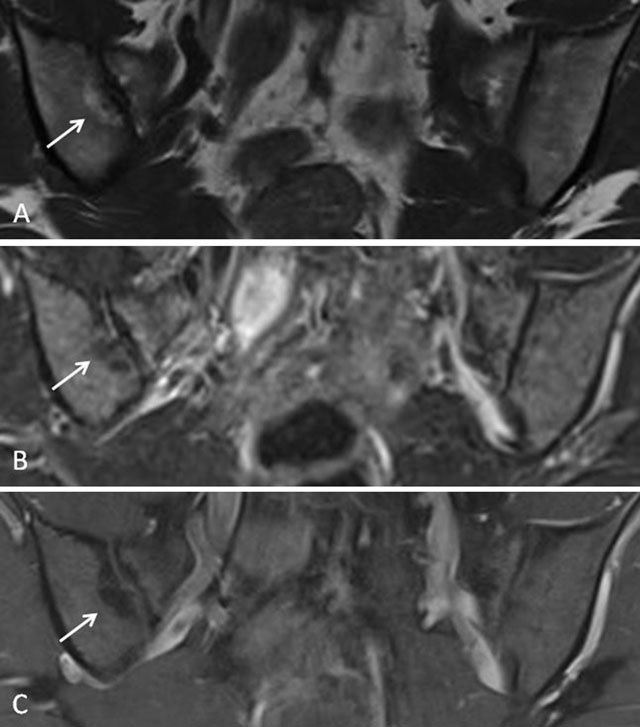
Structural lesions of sacroiliitis in a 17-year-old girl with JSpA. Semicoronal T1 (**a**), STIR (**b**), and contrast-enhanced fat-saturated T1-weighted (**c**) images show irregular delineation of the right SI joint with subchondral sclerosis and fat. There are also subtle erosions and subchondral sclerosis on the left side. No enhancement is seen.

Structural lesions are less frequently seen in children, but when present, they have a high specificity for JSpA, especially erosions and ankylosis (Figure 10) [21]. Erosions are—in contrast to adults—better seen on the STIR images and not on the T1-weighted images due to the ongoing ossification process. For the same reason, fat infiltration and sclerosis are not easy to recognize (Figure 11); our research indicates they are less important in children.

**Figure 12:**
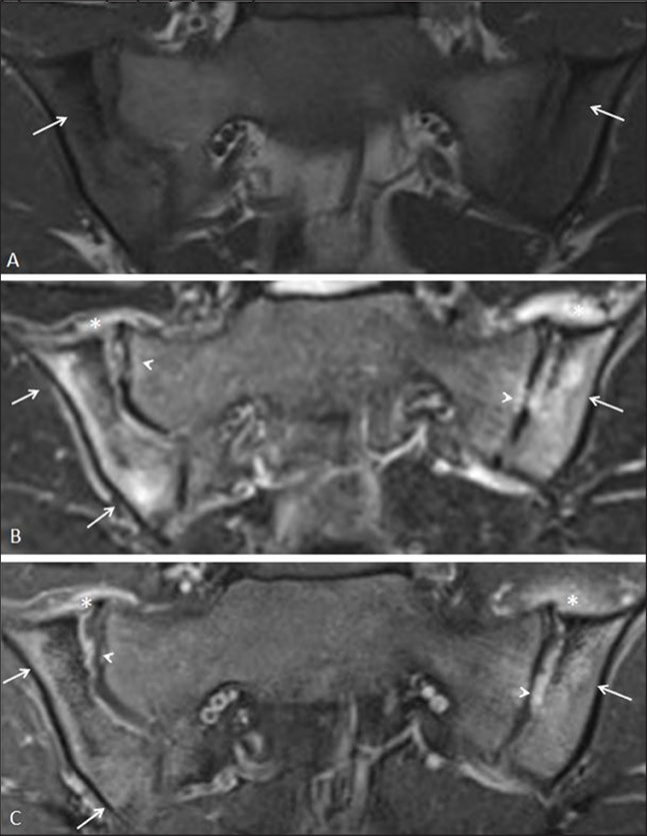
Multiple active and structural features of sacroiliitis seen on MRI in a 16-year-old girl with JSpA. **a.** Semicoronal T1-weighted image shows erosions and sclerosis in both sacroiliacal joints (arrows). **b.** Semicoronal STIR image shows high signal in the joint space of both sacroiliacal joints (short arrows) and bone marrow edema at the left iliac side (arrows). There is extensive capsulitis on both sides (asterisk). **c.** Corresponding contrast-enhanced fat-saturated T1-weighted image shows synovial enhancement in the erosions (short arrows) and enhancement at the site of the edema (arrows). Note also the enhancing capsulitis on both sides (asterisk).

**Figure 13:**
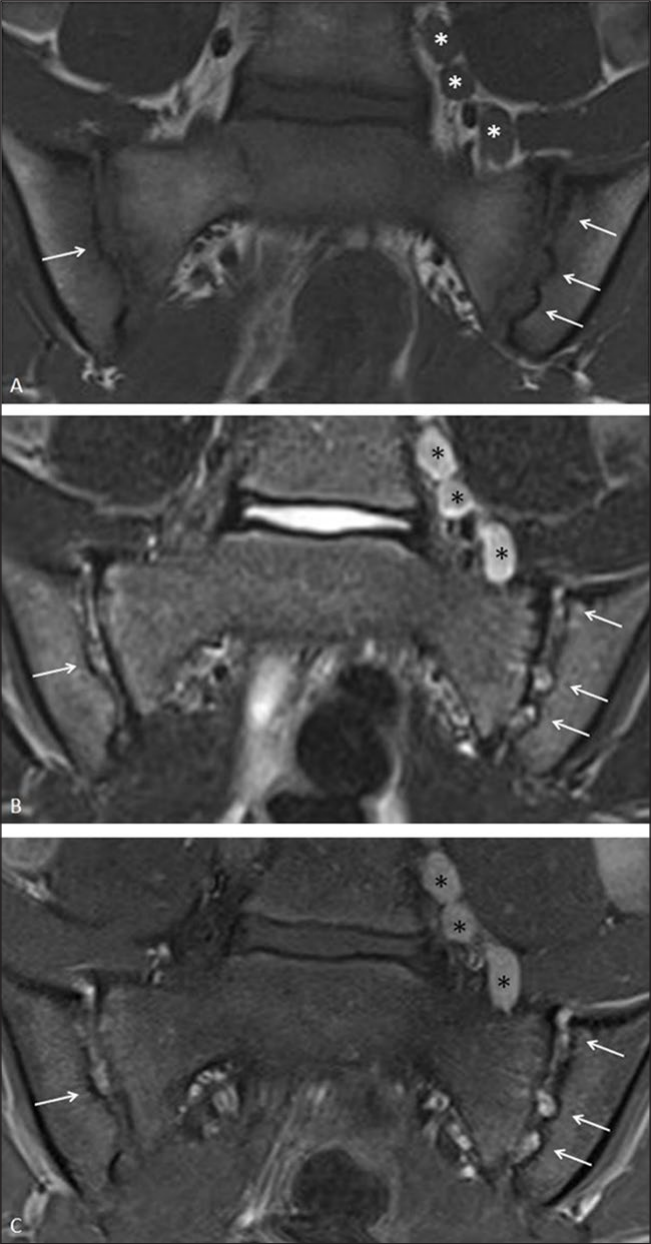
Active and structural sacroiliitis in a 17-year-old boy with JSpA. **a.** Semicoronal T1-weighted image show extensive erosions of the SI joints (arrows). **b.** Semicoronal STIR image shows high signal in the joint space and within the erosions (arrows). No surrounding bone marrow edema is visible. **c.** Contrast-enhanced fat-saturated T1-weighted image shows synovial enhancement in the erosions and the joint space. Note also the enlarged lymph nodes along the iliac vessels (asterisk).

**Figure 14:**
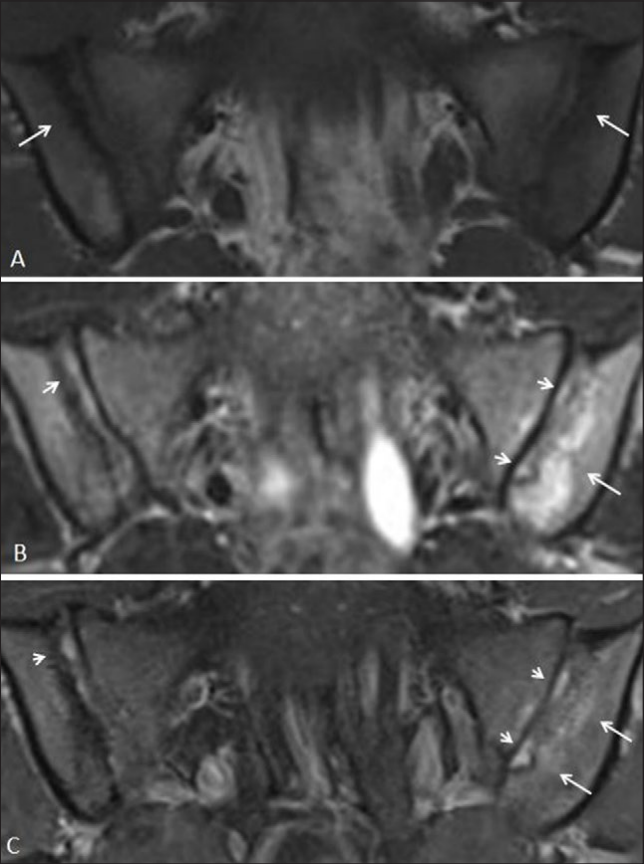
Active and structural sacroiliitis in a 15-year-old boy with JSpA. **a.** Semicoronal T1-weighted image shows erosions on both SI joints and subchondral sclerosis at the iliac side of the right SI joint (arrows). **b.** Semicoronal STIR image shows high signal in the joint space of both SI joints (short arrows) and bone marrow edema at the left iliac side (arrow). **c.** Contrast-enhanced fat-saturated T1-weighted image shows synovial enhancement in the erosions (short arrows) and enhancing osteitis (arrows).

**Figure 15:**
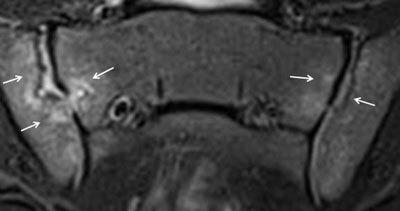
Active and structural sacroiliitis in a 14-year-old boy with JSpA. Semicoronal STIR image shows erosions of both the SI joints with surrounding bone marrow edema and correct signal in the joint space (arrows).

Luckily, in many cases, different features are seen concomitantly (Figures 3, 4, 12, 13, 14, 15), making it easier to diagnose sacroiliitis with more confidence. If BME is seen concomitant with synovitis, or erosions concomitant with BME or synovitis, the diagnosis of JSpA is very likely. Ankylosis, capsulitis, bone marrow edema, and erosion all have a high specificity for JSpA [21].

## Definition of Sacroiliitis on MRI

**Figure 16:**
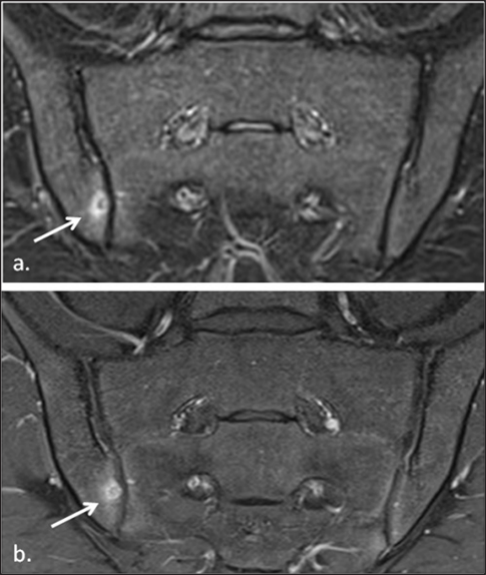
Active sacroiliitis in a 17-year-old girl with JSpA according to global assessment as well as to the ASAS definition of a positive MRI for sacroiliitis. **a.** Semicoronal STIR image shows an active lesion with BME at the iliac side of the right sacroiliac joint on two consecutive slides (only one slide shown) (arrow). **b.** Corresponding semicoronal fat-saturated T1-weighted image shows vivid enhancement of this site (arrow).

In the adult ASAS criteria, there is a clear definition of a positive MRI for sacroiliitis (Table 2) [15,16,17]. MRI is regarded positive for sacroiliitis if BME is clearly present and located in the typical subchondral or periarticular areas. If there is only one site of BME, this should be present on at least two MRI slices. If there is more than one signal on a single slice, one slice may be enough [15]. Other MRI features representing active inflammation of the SI joint, such as enthesitis or capsulitis alone, are not sufficient for a positive MRI for sacroiliitis; structural postinflammatory lesions in the sacroiliac joints, such as sclerosis, fat infiltration, erosion, or ankyloses, are not included in the definition [15,16,17]. We demonstrated that this adult ASAS definition can be applied in children (Figure 16), but the lower sensitivity limits its usefulness in daily practice (these results are not yet published).

**Table 2:** The ASAS Definition of Sacroiliitis on MRI [15,16,17].

Definition of Sacroiliitis on MRI (Positive MRI) as Applied in ASAS Classification*
Types of findings required for definition of sacroiliitis by MRI Bone marrow edema (BMO) (on STIR) or osteitis (on T1 post-Gd) highly suggestive of SpA must be clearly present and located in the typical anatomical areas (subchondral or periarticular bone marrow). The sole presence of other active inflammatory lesions such as synovitis, enthesitis, or capsulitis without concomitant BMO/osteitis is not sufficient for the definition of sacroiliitis on MRI. Structural lesions such as fat deposition, sclerosis, erosions, or bony ankylosis are likely to reflect previous inflammation. At this moment, however, the consensus group felt that the sole presence of structural lesions without concomitant BMO/osteitis does not suffice for the definition of a positive MRI. If an inflammatory bone marrow lesion appears to be present but it is hard to determine whether the lesion meets the criterion “highly suggestive for SpA,” the decision may be influenced by the presence of concomitant structural damage or other signs of inflammation, which in themselves do not suffice to meet the criterion. [update Lambert et al.]
Amount of signal required If there is only one signal (BMO lesion) for each MRI slice suggesting active inflammation, the BMO lesion should be present on at least two consecutive slices. If there is more than one signal (BMO lesion) on a single slice, one slice may be sufficient.

* Adapted from Sieper J, Rudwaleit M, Baraliakos X, et al., 2009; Rudwaleit M, Jurik AG, Hermann KG, et al., 2009; Lambert RG, Bakker PA, van der Heijde D, et al., 2016 [15,16,17].

**Figure 17:**
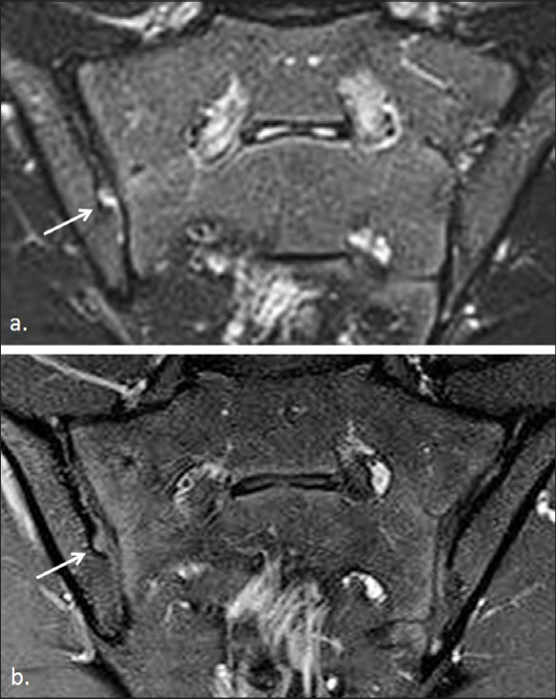
Active sacroiliitis in a 15-year-old boy with JSpA according to a global assessment of MRI for sacroiliitis, not according to the ASAS definition of a positive MRI for sacroiliitis. **a.** Semicoronal STIR image shows a focal nodular high signal in the right sacroiliac joint representing an active erosion (arrow). **b.** Semicoronal contrast-enhanced fat-saturated T1-weighted image shows corresponding enhancement of this lesion (arrow).

The main shortcoming of applying this definition is the fact that BME should be seen on two consecutive slices, which is often not the case in children (Figure 17). A possible explanation for this is the—in our experience—often less extensive BME, as mentioned above. Another factor that may contribute is the relatively smaller size of the SI joints in younger children, in whom the entire joint is only captured in a few 3-mm slices. BME in a single MRI slice obtained in a small child may represent a similar proportion of the joint as more than one slice of an adult patient. For this, thinner slices would be desirable, but thinner slices have technical issues. Thinner slices would produce more noise, resulting in a lower signal-to-noise (SNR) ratio, which is a measure for image quality that compares the level of a desired signal to the level of background noise. Especially for the STIR sequence, which is used for detecting BME, obtaining thinner slices is not possible, as this sequence already has a low SNR.

**Figure 18:**
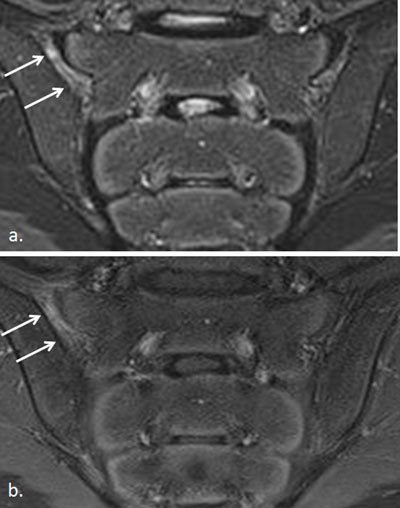
Active sacroiliitis with synovitis/retroarticular enthesitis in a 14-year-old boy with JSpA according to a global assessment of MRI for sacroiliitis, not according to the ASAS definition of a positive MRI for sacroiliitis. **a.** Semicoronal STIR MR image demonstrates synovitis/retroarticular enthesitis of the right sacroiliac joint (arrows). **b.** Semicoronal contrast-enhanced fat-saturated T1-weighted image shows synovial enhancement and enhancement of the retroarticular interosseous ligaments (arrows).

We made some suggestions to adapt the adult ASAS definition for a positive MRI to make it more pediatric-specific (Table 3). Including small BME lesions that are only visible on one slice or synovitis in the definition (Figures 17, 18) could increase the sensitivity without lowering the high specificity. However, this has some implications. For example, when a reader identifies BME on only a single slice, pitfalls such as vascular structures mimicking BME should be taken into account. Therefore, including CE-sequences can make it easier to diagnose osteitis with more confidence. Also, identifying the lesion on both paracoronal and axial sequences can help.

**Table 3:** Proposal for a Pediatric-specific Definition of Sacroiliitis on MRI.

Proposal for Definition of Sacroiliitis on MRI (Positive MRI) in Children
Types of findings required for definition of sacroiliitis by MRI Bone marrow edema (BMO) (on STIR) or osteitis (on T1 post-Gd) highly suggestive of SpA must be clearly present and located in the typical anatomical areas (subchondral or periarticular bone marrow). Synovitis with clearly enhancing synovium on T1 post-Gd in the entire sacroiliac joint (uni- or bilateral) The sole presence of other active inflammatory lesions, such as enthesitis or capsulitis without concomitant BMO/osteitis or synovitis, is not sufficient for the definition of sacroiliitis on MRI. Structural lesions, such as fat deposition, sclerosis, erosions, or bony ankyloses, are not sufficient for the definition of a positive MRI. If an inflammatory bone marrow lesion appears to be present but it is hard to determine whether the lesion meets the criterion of “highly suggestive for SpA,” the decision may be influenced by the presence of concomitant structural damage or other signs of inflammation, which in themselves do not suffice to meet the criterion.
Amount of signal required One BMO lesion on a single slice may be sufficient.

*Note:* The differences between the adult (Table 2) and the proposed pediatric-specific definition of sacroiliitis are marked in italics.

Capsulitis or retroarticular enthesitis alone seems not sufficient for diagnosing sacroiliitis, but it may influence the decision when other active features are present that in themselves are not sufficient to make a diagnosis of sacroiliitis on MRI.

## The Whole Picture

**Figure 19:**
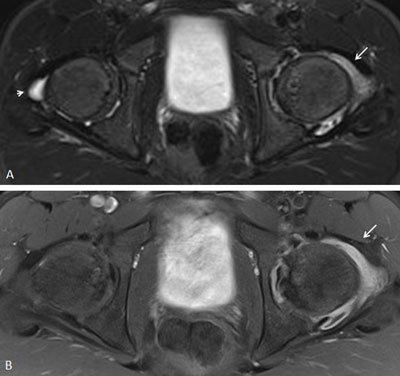
Hip arthritis in a 14-year-old boy with JSpA. **a.** Axial STIR image shows a joint effusion in the left hip joint (arrow). Note also the small amount of fluid in the right hip joint. **b.** Axial contrast-enhanced fat-saturated T1-weighted image shows synovial enhancement at the left hip joint (arrow). No enhancement is seen at the right hip joint.

**Figure 20:**
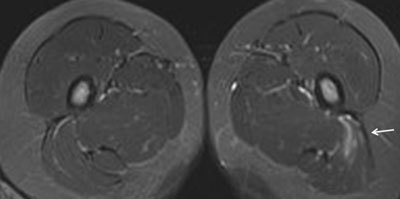
Axial STIR MR image in an 8-year-old girl with enthesitis-related arthritis shows enthesitis of the left gluteus maximus insertion (arrow).

**Figure 21:**
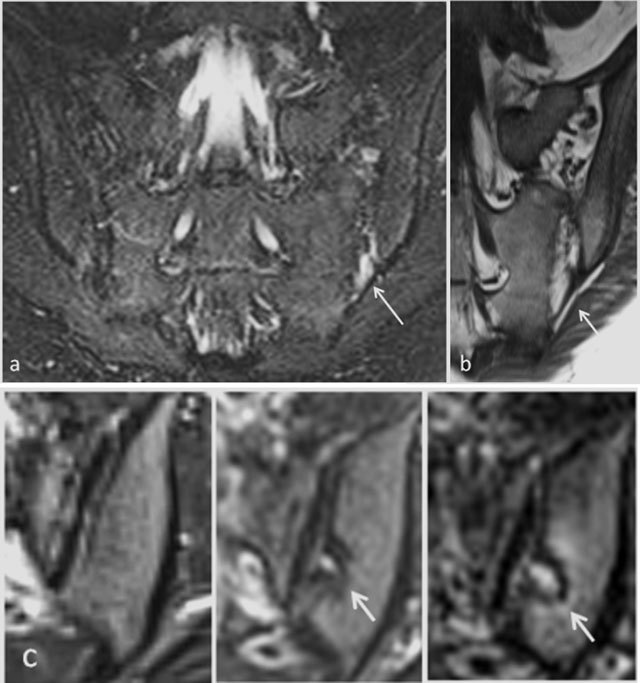
**a.** Paracoronal STIR images in a 16-year-old girl with enthesitis-related arthritis demonstrates a left-sided enthesitis of the retroarticular interosseous ligaments as only finding at the first MRI of the sacroiliac joints. **b.** T1 image demonstrates that the high signal is seen in the retroarticular fat tissue and not in the cartilaginous part of the SI joint. **c.** Paracoronal STIR image at first MRI, two and three years later. Follow-up MRI in this patient shows formation of a small erosion at the iliac side of the left sacroiliac joint, evolving to a large erosion three years later.

**Figure 22:**
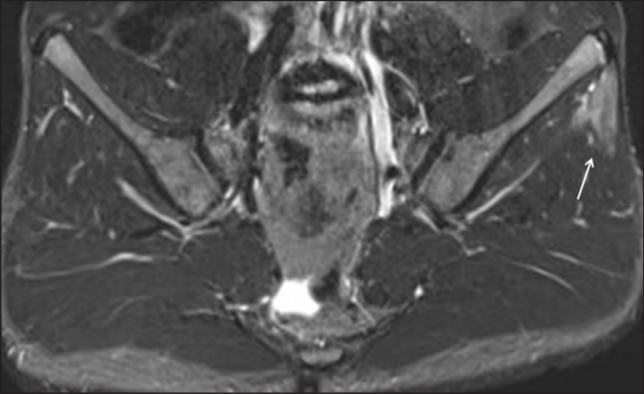
Semicoronal STIR MR image of the SI joint in a 15-year-old boy with arthralgias (ERA-negative) shows edema at the origin of the gluteus medius muscle at the iliac crest representing enthesitis.

**Figure 23:**
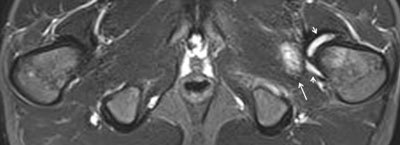
Axial STIR image in a 14-year-old boy with enthesitis-related arthritis shows high signal intensity in the left obturator externus muscle (arrow), representing hip enthesitis. Note also some fluid in the left hip joint (short arrows).

**Figure 24:**
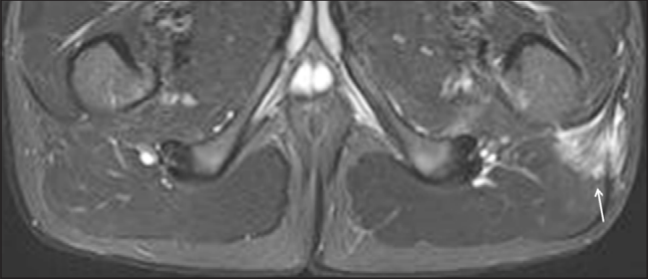
Axial STIR image in a 15-year-old boy with enthesitis-related arthritis demonstrates enthesitis of the left gluteus maximus insertion (arrow).

**Figure 25:**
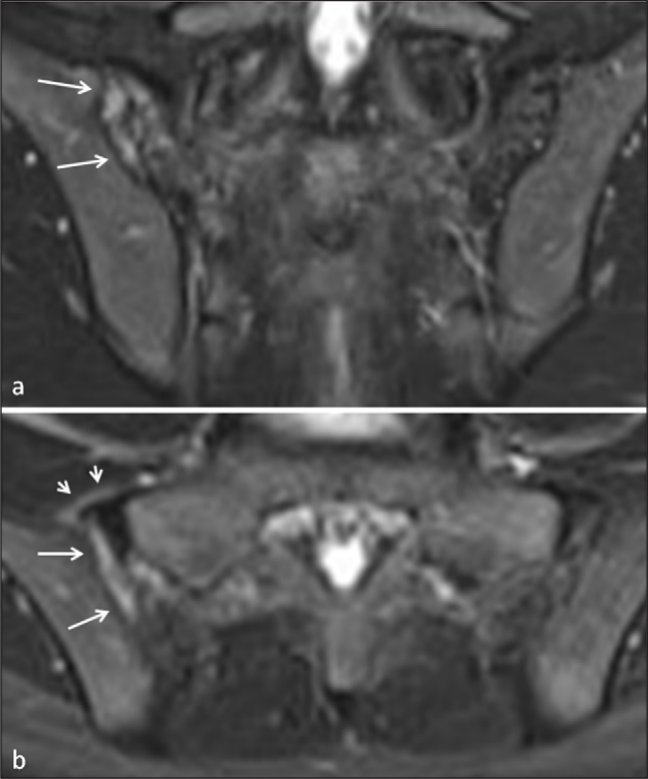
**a.** Semicoronal and **b.** axial STIR MR image in a 12-year-old boy with enthesitis-related arthritis demonstrates enthesitis of the retroarticular interosseous ligaments on the right side (arrows). Note also the sacroiliitis with fluid and capsulitis at the right SI joint (short arrows).

When imaging sacroiliac joints on MRI, other structures than the sacroiliac joints can be evaluated as well, such as the hip joint (Figure 19) and the pelvic entheses (Figures 20, 21, 22, 23, 24, 25), especially on the axial images [23]. Hip joints are frequently involved in juvenile SpA, as JSpA, in most cases, presents with arthritis/enthesitis of the lower extremities [1,2]. Enthesitis is a primary clinical criterion in enthesitis-related arthritis (ERA). The diagnosis is based on palpable tenderness at insertion sites alone [3]. Enthesitis is not always easy to assess clinically, especially deep-seated entheses such as the pelvic entheses are difficult to examine. MRI is excellent for demonstrating enthesitis, depicting not only bone marrow edema but also soft tissue inflammation and joint effusion/bursitis [4], as has been shown in adult SpA patients [4,15].

In children with ERA, however, it has been shown that the number of active entheses and joints at onset can predict sacroiliitis at follow-up [19]. Assessment of entheses on pelvic MRI can therefore be of great value. In contrast to adult studies, we found pelvic enthesitis not to be very sensitive for JSpA, but we did find a high correlation with sacroiliitis [23]. At the moment, enthesitis is only a clinical criterion. However, in our study, some patients could have had a change in diagnosis if pelvic enthesitis seen on MRI was included in the criteria [23]. Still, it indicates active ongoing inflammation and may play a role in assessment of the inflammatory status of the child and should be carefully sought and noted by radiologists examining MRI of the sacroiliac joints in children.

## Conclusion

MRI of the sacroiliac joints in children is a useful tool and should be applied in clinical practice in children suspected for JSpA. There are multiple features of active inflammation and structural damage visible on MRI of the sacroiliac joints that can provide a specific diagnosis of JSpA when present in children with suspected sacroiliitis. Luckily, in many cases, different features are seen concomitantly, making it easier to diagnose sacroiliitis with more confidence.

The adult ASAS definition for a positive MRI needs some adaptations for children. Including small BME lesions that are only visible on one slice or synovitis in the definition could increase its usefulness in daily clinical practice.

MRI without contrast administration is sufficient to identify bone marrow edema, capsulitis, and retroarticular enthesitis as features of active sacroiliitis in JSpA. In selected cases, when high short tau inversion recovery (STIR) signal in the joint is the only finding, gadolinium-enhanced images may help to confirm the presence of synovitis.

We found a high correlation between pelvic enthesitis and sacroiliitis on MRI of the sacroiliac joints in children. As pelvic enthesitis indicates active inflammation, it may play a role in assessment of the inflammatory status. Therefore, it should be carefully sought and noted by radiologists examining MRI of the sacroiliac joints in children.
